# The Impact of the COVID-19 Pandemic on Independent Creative Activities in Two Large Cities in Romania

**DOI:** 10.3390/ijerph18147674

**Published:** 2021-07-19

**Authors:** Nicolae Popa, Ana-Maria Pop, Alexandra-Camelia Marian-Potra, Pompei Cocean, Gheorghe-Gavrilă Hognogi, Nicoleta Afrodita David

**Affiliations:** 1 Department of Geography, Faculty of Chemistry, Biology, Geography, West University of Timișoara, 4 Vasile Pârvan Boulevard, 300223 Timișoara, Romania; nicolae.popa@e-uvt.ro; 2 Centre for Regional Geography, Faculty of Geography, Babeș-Bolyai University, 5-7 Clinicilor Street, 400006 Cluj-Napoca, Romania; ana-maria.pop@ubbcluj.ro (A.-M.P.); gheorghe.hognogi@ubbcluj.ro (G.-G.H.); nicoleta.david@ubbcluj.ro (N.A.D.); 3 Cluj-Napoca Branch, Romanian Academy, 42 Treboniu Laurian Street, 400271 Cluj-Napoca, Romania; pompei.cocean@ubbcluj.ro

**Keywords:** creative industries, independent cultural sector, social resilience, COVID-19 pandemic, Romania

## Abstract

The COVID-19 pandemic has had both financial and activity-related effects on a number of areas of activity, among which those involving the creative industries have proved to be weak in their capacity to survive the halting of all events held in physical spaces. The long-term effects of the current health crisis are bringing about changes in cultural demand and offer and highlighting the need to adapt and to think of new ways of functioning. Taking its cue from this situation, the research underlying our article set out to investigate the ways in which Romania’s independent creative sector is adapting. We achieved this by means of conducting 25 semi-structured interviews and undertaking case studies of two cities that are among the most effervescent from the point of view of cultural and creative industries, Timișoara and Cluj-Napoca. With the strengthening of this sector as the aim in view, the forms of early social resilience we identified are capable in the short term of taking action to ensure the survival of some of the spaces; in the medium term, through activating mechanisms that encourage entrepreneurial spirit, they will be able to adapt to any external shock.

## 1. Introduction

The COVID-19 pandemic has affected all areas of socio-economic life on a global scale and in an unprecedented way. Many governments in all parts of the world have imposed local lockdowns with the aim of encouraging people to socially distance. The very widespread application of measures of this kind has meant that all activities judged to be non-essential have been almost completely stopped [1]. This has led to technical unemployment and to an increase in bankruptcies, particularly among small and medium-sized businesses, and subsequently also to an increase in real unemployment, which has nourished fears of a large-scale economic recession or even of an eventual wholesale economic collapse [2].

After the initial shock, which took the whole world by surprise and led to extremely stringent lockdowns in many countries in Europe, governments began to respond in more nuanced ways [3]. Sectors key to the proper functioning of the economy and to the safety of society were kept functioning, with careful implementation of social distancing measures and with hygiene being guaranteed throughout the organizational and technological stages involved [1]. By contrast, sectors regarded as less vital and less capable of operating without overcrowding and extensive human contact remained closed or were partially reopened if the local level of SARS-Cov 2 infection permitted this [4]. For example, in the second quarter of 2020, in the 20 EU countries for which Eurostat data were available, the number of passengers traveling by air fell by between 96% and 99% compared with the same period in 2019 [5]. In Romania, the independent creative cultural sector was also severely affected.

Following the fall of Romania’s communist regime, the public expression and perception of the creative industries were stimulated by the increasingly frequent opportunities for creatives to establish international contacts that came once the country had joined the EU (in 2007). The expansion took place mainly between 2015 and 2019 in her large cities, multi-faceted university centers with high-level training programs, a tradition in the artistic and informatics areas, an abundance of young people, and local environments that were cosmopolitan and offered fertile ground for the cultural and creative industries to thrive: Bucharest Cluj-Napoca, Timișoara, and Iași, followed by Craiova, Sibiu, Brașov, Constanța, and others.

Our research is interesting because it addresses an emerging sector, the independent cultural and creative sector, from an emerging EU country, Romania [6], whose regional urban poles have become outstanding competitors of the capital and are operating more and more freely and more internationally. In fact, Cluj-Napoca and Timisoara are the strongest regional cities in this country.

This being the background, we set ourselves the task of studying the reaction of the independent cultural and creative sector in the face of the unprecedented crisis that broke in 2020, in which, in order to survive, companies and creatives have been compelled to change the ways in which they express themselves, make their products known, and exploit possibilities. What now determines the success or failure of independent sector creative industries is not so much the nature of the environment in which they have found a niche—particularly if support from that environment is late in coming—but more the extent to which the entrepreneurs concerned manage to reinvent themselves to meet the needs of a changing client base [7]. This is also the raison d’être of the present study, the purpose of which is to investigate the independent creative sector in the cities of Cluj-Napoca and Timișoara from the perspective of the way they have reacted to the shock of the COVID-19 pandemic and their success or otherwise in adapting to the new situation that governs functioning and social interaction.

What we are setting out to do in this article is to analyze the impact of the COVID-19 pandemic on the independent sector of Romania’s cultural and creative industries, since we are starting out from the hypothesis that this is extremely vulnerable in the face of lockdown and of the various kinds of restrictions that had been introduced up to the point at which we concluded our information gathering. The independent cultural and creative sector is a spontaneous, diverse phenomenon whose precise boundaries are as yet unclear. Both its interconnections with different sectors of activity and the nature of the forms of organization and expression that characterize it [8] make it hard for it to be regulated or fitted into a framework without this affecting its spontaneity [9]. This unique character, the independent sector’s strongest point in times of economic expansion, is also its point of greatest vulnerability in crisis circumstances, when the legally guaranteed public funding system is demonstrably insufficient and ill-adapted to meet its needs.

The specialist literature has managed to reach a degree of consensus regarding a general definition [10], with contributions ranging from synthetic-type academic studies of its beginnings [11,12] to older [13] and more recent official definitions that serve as a basis for programs for the development of this sector (such as the Creative Europe Programme, which was relaunched by the European Commission in 2018).

The specialist literature devoted to creativity does in fact frequently make a distinction between “Big C” and “little c” creativity. “Big C” creativity refers to the highest levels of creativity, as illustrated by unique, unmistakable creators who represent an elite minority of geniuses who exist at one remove from society [14]. By contrast, little c creativity refers to the everyday creative potential that most people possess; this is a quality essential to human development and a vital component of a happy and healthy life [15]. In this article, we insist on the impact of the pandemic on the independent cultural and creative sector, whose activities fall rather into the second type of creativity (little c).

The cultural and creative sector is important for the balance and resilience of society as a whole, and creativity may be seen as a socio-cultural act that can be distributed between actors and multiple elements [16]. It contributes to the well-being of the population in general [17], from the professional and economic flourishing of the creators and distributors of cultural–creative products, together with that of the multipliers of creativity, to the increasing of their degree of satisfaction with life on the part of the consumers of culture [18].

The most serious weakness of the cultural and creative industries is that they are regarded by society as a domain whose survival in times of crisis is not absolutely essential. A further specific feature of many cultural and creative industries is that they flourish most when in direct contact with the consumer, in a context of relaxation and enjoyment. Online possibilities do exist, and extensive use has indeed been made of them during the pandemic [1], but in a world that has been forced to move to digital in many areas vital to economic security, online consumption of culture does not satisfy the need for relating socially.

Adapting to critical events, from natural disasters and epidemics to economic recessions, calls for optimal management of their effects via the identification of solutions and the development of models of resilience [19,20], such as the PEOPLES model [21], based on the principle of the 4R (reduction, readiness, response and recovery). Our research—conducted in the midst of the COVID-19 pandemic—does not allow us to cover all stages of the model, but captures the immediate, quasi-spontaneous reactions of ICCS actors to the shock of pandemic restrictions.

The identification of methods of helping the independent cultural–creative sector to regain its balance draws on the concept of social resilience [22,23]. This is concerned with collective methods of adapting to and finding optimal ways to handle critical situations and depends on social unity within a group [22,24]. Social resilience is a process of strengthening abilities [25] and also functions as a social security network based on mutual aid [26]. Cutter et al. posit a link between social resilience and cognitive indicators such as individuals’ attitudes, values, and beliefs and also their self-perceptions and views about their own development environment [27].

What functions as the essential criterion for the resilience of the independent cultural-creative sector in the current pandemic is its entrepreneurial ability to create something from necessity within a short time [7], together with the creation of or joining of cultural associations and networks, which is seen more as a source of reliable support in situations of risk [28].

The work of the cultural–creative sector largely revolves around a calendar of events, particularly art fairs, exhibitions, performance art events, and training courses [29]. The transferal of these activities from the offline to the online environment via the use of social media has been one of the resilience measures adopted during the pandemic by numerous actors in the independent cultural–creative area [30]. Digitalization is simple to implement; however, it is not capable of providing all cultural and creative activities with a firm foundation.

## 2. Materials and Methods

The 2019 EU report The Cultural and Creative Cities Monitor lists six cities in Romania among its 190 cultural–creative European cities [31]. For the present study, we have chosen two of these, Timișoara and Cluj-Napoca. Both are (a) major demographic basins with over 350,000 inhabitants, representing a substantial social capital; (b) cities with a prestigious university tradition, with specializations that allow students to graduate in a number of areas of culture (drama, visual arts, photography, design, translation studies, etc.); (c) communities that submitted a multi-faceted cultural program as part of their entries for the European Cultural Capital contest (a role won by Timișoara and due to be implemented in 2023) (d) centers that possess diverse and complex official cultural spaces and independent creative entities.

Against the background of the non-existence of an integrated database of the Romanian independent cultural sector, in the period 2019–2020, we performed an up-to-date radiograph of the creative and cultural spaces in Cluj-Napoca (n = 82) and Timișoara (n = 59). This allowed us to have an almost exhaustive representation of the structure and pre-pandemic dynamics of ICCS. As methodological tools, we used survey sheets (n = 141) but also interviews (n = 64). The selection of qualitative methods is justified by the fact that independent cultural entities do not have an institutionalized character; the lapidary data found in their address, where appropriate, are based on statements on the responsibility of cultural operators. In most EU Member States, artists, cultural workers, and creative workers do not have a legal status [32]. Recognizing their legitimacy would be a first step in accessing a range of official quantitative information associated with the profile of each space.

Taking into account the vulnerability of cultural and creative spaces induced by the COVID-19 pandemic, we carried out a spatio-temporal assessment of the perceptions of the managers of some cultural entities in Romania, covering a range of cognitive indicators [27], in order to identify their forms of adaptation. The analysis had the following features:

- Applying eight-item semi-structured interviews to the managers of creative spaces in Timișoara and Cluj-Napoca (n = 25) in the period 1 October–17 November 2020 (Table 1). The selection of these creative and cultural spaces was made by referring to the ICCS database we created previously, taking into account the representation of most existing types of creative spaces, the forms of adaptation identified in the online environment during the state of emergency, and the collaboration of some of the managers of creative spaces.
- Filtering participants’ perceptions by relating them to two critical periods of the COVID-19 pandemic, the state of emergency (16 March–14 May 2020) and the states of alert (starting on 15 May 20, until 15 November 2020, when interviewing stopped), both with reference also to the cultural activities that were taking place before the pandemic was declared.

The cultural and creative spaces identified at the level of the two urban centers analyzed are predominantly part of the category of community spaces. A major part of them do not have as purpose activities with profits, especially since some of them do not have a legal form; instead, they represent communities of freelancers or even civic initiatives (Someș Delivery, Căminul Subcultural etc.). As regards the maker spaces, business accelerators, or hubs, they work by selling the products created, their activity being closely related to the entrepreneurial openness of administrators/managers. The recognition of independent artists cannot be defined only in economic terms, the activity performed by those working in the cultural and creative sector being a process and not just a tangible or produced object [31].

Our investigation of ICCS resilience in the two cities during the pandemic also covered the dimensions of social resilience summarized by Keck and Sakdapolrak [22]: (1) Coping capacities—the ability of social actors to cope with and survive adversities of all kinds; (2) Adaptive capacities—their ability to learn from past experiences and to adapt to future challenges in their day-to-day lives; (3) Transformative capacities—their ability to create sets of solutions capable of promoting individual well-being and a more robust approach to future crises.

## 3. Results

### 3.1. The Independent Cultural Sector in Romania on the Threshold of the COVID-19 Pandemic

The specialist literature captures the variety and diversity of initiatives in the independent cultural–creative area [8,33]. In the older EU members in the center and west of the continent, the creative sector has a more coherent basis in law and official status and is more fully documented than is the case in the countries that joined after the fall of the Iron Curtain. In the latter, we still find a public state-supported cultural sector that is stable and well regulated, while the independent creative sector is spontaneous in nature and operates on the basis of legislation covering small and medium-sized companies and authorized freelancers.

In Romania, the cultural institutions that are officially recognized at the national level are public bodies and all come under the Ministry of Culture. By contrast, the independent cultural sector is not institutionalized. The independent cultural spaces that have appeared during the past 20 years have arisen spontaneously, particularly in the country’s large university centers, which themselves possess a large number of companies operating in the creative industries sector. In terms of legal status, cultural bodies, whether involving freelancers or employees, fall into a number of different categories of juridical persons: the majority are NGOs (in the form of associations and foundations), followed by limited companies (some of which started as start-ups in the creative industry area), independent artists (with or without authorized freelancer status), and even groups that take the form of informal communities that bring together people with common interests.

**Figure 1:**
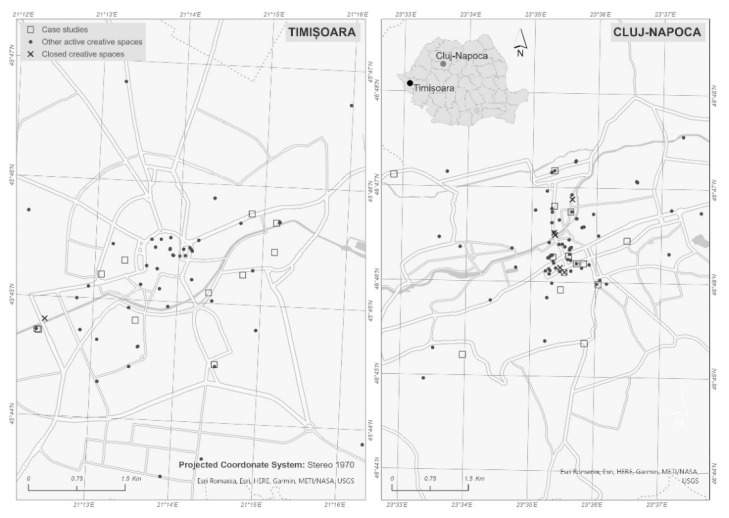
Location of creative and cultural spaces in Timișoara and Cluj-Napoca (2020).

In the absence in Romania of any centralized databases recording these independent cultural spaces, our investigation of the ICCS in these two cities is based on field research carried out in the period March to November 2020. Taking as our starting point the typology suggested by Montalto et al. [31], all the spaces identified were assigned to at least one of the types proposed. A total of 59 creative spaces were identified in Timișoara, and 82 were identified in Cluj-Napoca (Figure 1).

Among the sectors most deeply affected is that of the cultural and creative industries [2]. The extent to which it has been paralyzed has differed from country to country, but there have been effects on a number of levels, depending on the way the industry concerned was run, the content of its activities, and the nature of the relationship with the target audience [29].

It was only in the second half of 2020 that the National Institute for Research and Cultural Formation set up the Cultural Register as an initial response to the lack of a centralized database of people working in the field of culture in Romania. This involved the mapping, country-wide, of 6606 cultural workers (individuals), 1179 cultural NGOs, and 3135 commercial companies, covering all the domains of artistic and cultural activity (theater, arts, visual arts, books, patrimony, audio-visual) [34,35]. The majority of spaces fall into the category of community spaces, as is also the case with most of the cultural spaces selected as case studies for the purposes of this study; maker spaces and coworking spaces are next in frequency.

The EU has been displaying an increasing interest in promoting cultural and creative spaces (CCS), which in the last thirty years have been supported via dedicated funding programs: Media (1991–2013), MEDIA Mundus (2011–2013), Culture (2000–2013), and Creative Europe (2014–2020), the last mentioned offering guarantees and loans to all small and medium-sized enterprises in the cultural and creative areas. However, in Romania, the funding received through the programs to which the country gained access after joining the EU has been devoted exclusively to its cultural heritage, without any financing of projects in the area of developing the cultural infrastructure or giving support to cultural and creative services. Nevertheless, there has been progress. CCSs were one of the ten sectors identified as priorities by the National Strategy for Competitiveness for 2014–2020, while for the 2020–2027 funding cycle, the cultural and creative sector is one of the priority areas both for the EU and for Romania.

The summary of the SWOT analysis of the Romanian creative and cultural sector, which we conducted before the pandemic, highlights that the cultural and creative sector in several large cities in Romania has advantages that can compete with any other international urban center of comparable size (Table 2). The opening of local and regional actors involved in the creative industry and cultural field to internationalization, visibility, tolerance, innovation, which is materialized by integrating most cultural spaces in different forms of association/collaboration, is part of a natural trajectory of involvement in creating and implementing cultural policies meant to change the perception of the artist status. Beyond the vulnerability of the artists’ and cultural workers’ status and even the precariousness of the locations where some of them work, in the two urban centers investigated, Timișoara and Cluj-Napoca, there is an individualized presence of a constant and continuous training environment for professionals in the cultural and creative sector that maintains a complex, diversified cultural program with international recognition.

As regards the documenting of this area, the first move to gain information regarding the situation and development of the creative domain in Romania took the form of projects that made use of OMPI/WIPO methodology and a formula based on authorial rights: The economic contribution of copyright-based sectors in Romania, 2002–2005 (CSCDC, 2007) and The contribution of copyright-based industries to the national economy for the period 2006–2009 (CCCDC, 2011), which were co-financed by the European Social Fund, POSDRU, 2007–2013. Since then, official reports and academic studies have mushroomed, while however remaining modest in number, although the cultural–creative area has seen a gradual expansion (Table 3).

According to Cultural and Creative Cities Monitor (2019) data for the EU, the two cities assessed in this article rank 27th (Cluj-Napoca) and 35th (Timișoara) on the Cultural Vibrancy performance index; Cluj-Napoca ranks 9th for Creative Economy, while Timișoara is 17th for Enabling Environment. These rankings take account of 40 EU cities with populations of between 250,000 and 400,000.

### 3.2. Ways in Which Creative and Cultural Spaces (SCCs) Have Met with Obstacles and Have Adapted during Lockdown

Increasing numbers of cases of the COVID-19 virus prompted the Romanian government to impose a two-month state of emergency (16 March–14 May 2020). A number of government decisions (Decision no. 6/6 March 2020; Decision no. 11/13 March 2020) and military ordinances (Military Ordinance no. 1/17 March 2020; Military Ordinance no. 2/21 March 2020), which began to be issued as soon as the first case in Romania was officially declared (on 27 February 2020) brought into force a range of social distancing measures which affected, among others, those working in the area of culture. Among these, we may mention the restricting of cultural–artistic activity and a forbidding of activities involving the public that led in fact to the halting of all activities and thus by implication of cultural ones. Slight moves in the direction of relaxation, which found concrete expression in a gradual resumption of activity on the part of some cultural bodies and involved the observing of rules regarding prevention and protection, appeared only after the state of emergency had been succeeded by a state of alert (through Decision no. 24/14 May 2020).

The non-existence of any regular income, a consequence both of the nature of the activities that normally take place in the cultural spaces (hiring out of halls and event venues, staging/hosting of studios/workshops/performance events) and of the fact that the majority of the initiatives involved are non-profit organizations, meant that during lockdown, it was impossible to provide the artists and staff involved with a regular income. As a part of a uniform whole-country approach to addressing the effects of the measures taken to limit the spread of the virus, the central government decreed the following types of support: access to a range of grants as established by Emergency Ordinance no. 130/2020; micro-grants of 2000 euros, available only to cultural workers who figured in the CULT Register; the technical unemployment scheme (75% of the national mean gross salary); and compensation for authorial rights for people whose income was derived from the sale of copyrights. While at the individual level, some kinds of subsistence support did exist during the pandemic, the perception of those working in the area of culture is that where organizations were concerned, these kinds of support were not sustainable; they were decided upon under time pressure and were not part of any settled long-term cultural policy (I1, I4, I6, I14, I19, I22).

“*The authorities did not help us much as an organization. There were no initiatives designed to support the creative area. Some money was promised but nothing arrived. And it would have made a significant difference, because many of the themes of today’s world are discussed in the independent creative environment. The support we receive from the authorities goes no further than fine words. There are no coherent long-term cultural strategies as there are in France or Germany.*” (I6, Basca, Timișoara).

At a local level, in the two cities studied, Timișoara and Cluj-Napoca, depending on the financial capital of each city and also on initiatives taken to support the cultural program in recent years, opportunities for cultural operators to provide help to one another found individual expression in the launching of local contests (one example being the Culturpreneurs programme initiated by the Cluj Cultural Centre in partnership with Cluj-Napoca City Hall). In addition to this, the fact that some bodies have been supported by private sponsors has provided them with a temporary but useful way to continue to operate in the same spaces; examples would be the Interest Centre, supported by its official partner, Banca Transilvania, and Solid Arts and Entertainment in Timișoara, supported by Kaufland in accordance with the contract between them. However, many of the respondents interviewed (I5, I10, I12, I15, I16, I17, I20, I24) had not requested/enjoyed any kind of support either from the authorities or from the private sector.

The canceling of events and the barring of the public from creative spaces meant that the managers of creative spaces were forced to face up to a new context involving major challenges to viability and functionality. For some of them, the immediate reaction was to close the spaces concerned (n = six creative spaces closed, five of them in Cluj-Napoca and one in Timișoara) (Figure 1).

A comparative analysis of the situation prior to the pandemic vis-à-vis that during the state of emergency and immediately after it, as viewed through the reflections of the creative sector workers who were our interview respondents, threw into relief the fact that the picture was a complex one, with interviewees reporting different perspectives:

- A generally better situation, reported by cultural operators who benefited from additional alternative sources of funding (Interest Centre, Muzeon, Cowork Timișoara);
- A situation that was better in some ways, since it drove them to communicate and develop solidarity (INCUBOXX, Multiplexity, Plan Zero);
- A generally more difficult situation, with long-term effects in the absence of a regular cultural program (Digital Canvas, Create.act.enjoy, Loc în spațiu (Place in Space), MATCA artspace, Music Hub, ParaPark, Reactor de creație și experiment (Creation and Experiment Reactor), RoCreator, Smash Studio);
- A situation that was less favorable in some ways, the aspects most frequently cited being the absence of the public, a reduction in the number of events, and the impossibility of predicting the spread of the pandemic and the restrictive measures that would be imposed (Casa cu Iederă (Ivy House), Solid Arts and Entertainment, Atelier Patru (Atelier Four), Milestone, MindsHub);
- Instances of bankruptcy (Dive, Magic Puppet, etc.).

A national opinion survey carried out on a representative sample of people on the subject of accessing culture in the context of the COVID-19 pandemic highlighted a trend toward internet use in the second six months of 2020 [35]. Moving wholly or partially to the online environment was also reported by respondents to our interviews, in percentages that ranged from 10–25% for maker spaces and coworking spaces to 80–90% for those angled toward digital creation, visual arts, or design. Additionally, reluctance to participate in cultural events, in the general context of insecurity created by the perceived lack of stability of the living environment, is a first indication of a change in public mentality, as brought out by our research also:

“*Given the situation we are in, even if the spaces were to reopen tomorrow the public would not come [...] We have had events that were free and people didn’t come*” (I16, Cluj-Napoca).

Some of those interviewed were harshly critical of the toughness of the restrictions imposed during the state of emergency on the grounds of their very serious economic and social impact, all the more so since the lockdown proved to be incapable of combating the spread of the virus:

“*Economically speaking, making the country grind to a halt was extremely damaging and didn’t even solve the problem of the pandemic! Culture, tourism, sport, and events all had to endure the kind of violent suppression that I am not in agreement with, because it doesn’t produce results, either in Romania or in other countries!*” (I3, Casa cu iederă, Timișoara).

### 3.3. To Be or Not to Be Resilient?

The cultural sector, along with other areas of activity throughout the world, was seriously impacted [36], firstly by the COVID-19 pandemic itself, in that some people became infected, and secondly by the measures taken to limit the spread of the SARS-Cov-2 virus, which are measures that produced a reaction, both individual and collective, from the categories of people affected.

The views of our 25 interviewees, all businessmen/women and/or managers of independent cultural spaces in Timișoara or Cluj-Napoca and active members of the creative industry sector, show very clearly at a local level the changes that took place and by implication the kinds of action those cultural operators and entrepreneurs who create events for the wider public resorted to. We may observe a gradual shift from reactions that are passive and inactive and show a dependency on the decisions of higher authorities to reactions of adaptation, both by taking a stand and campaigning for support for the needs of independent artists and by restructuring activities:

- Stopping some physical space activities and relocating them in the online environment (INCUBOXX, Solid Arts and Entertainment, Create.act.enjoy, Music Hub, Milestone) or even conceiving new ones designed specifically for the digital environment (Digital Canvas, Magic Puppet, ParaPark, Reactor de creație și experiment, Smash Studio, Zug.zone, Cowork Timișoara, MindsHub);
- Creating offline events once the shift from the state of emergency to the state of alert had brought a degree of relaxation (INCUBOXX, Casa cu Iederă, Centrul de Interes, Create.act.enjoy, Loc în spațiu, Muzeon), but limiting access:
  “*We’ve begun operating, but everything is extremely frustrating, you can’t foresee anything, you don’t know which collaborators you can rely on long-term, what public you will get... everything keeps changing from one day to the next! There are also issues of profitability: our hall has a capacity of 50 but we can’t admit more than 15 people. It’s very frustrating for the artists too: being online and only online is very frustrating! Drama is intrinsically connected with being physically present!*” (I6, Basca, Timișoara)
- Participating in open-air festival events (Solid Arts and Entertainment, Magic Puppet, MATCA artspace, Reactor de creație și experiment, Cowork Timișoara);
- Developing new cultural products, social in character, that support the health environment (Create.act.enjoy);
- Community development and rethinking the public (INCUBOXX, Create.act.enjoy).
- The need to understand and sometimes even to perceive the cultural sector as a vulnerable community was raised by many independent artists, with a number of petitions on this topic being launched nationally (n = 19 public documents). One such collective issue is the dichotomy between public (state) cultural spaces and independent ones. This has been pointed out many times in public discourse, reference being made to inequalities in funding received, government support, and access to digital technology and to the cultural infrastructure (I16, I22).

When questioned about a pessimistic scenario involving the reintroduction of a state of emergency, respondents reacted in similar ways, speaking about continuing to work remotely (INCUBOXX), rethinking over 75% of their activity for an online format, both in Timișoara and in Cluj-Napoca (INCUBOXX, Digital Canvas, Solid Arts and Entertainment, Centrul de Interes, Create.act.enjoy, Loc în spațiu, ParaPark, Reactor de creație și experiment, Smash Studio, Zug.zone, Plan Zero, TeMiriCe (YouWonderWhat)), and the developing of new cultural concepts (Multiplexity, Digital Canvas, Music Hub).

However, other freelancers and managers of creative spaces were hesitant about continuing to operate online; this reluctance stemmed from the high level of interaction ideally involved, for example in the case of performance arts (theater, dance). This category of interviewees underlined as disadvantages the lack of immediate contact with the public, the absence of real feedback, the disappearance of the possibility of conveying and perceiving emotion (Casa cu Iederă, Dive, Magic Puppet, Create.act.enjoy), and online overload:

“*People are already super-saturated with online stuff. They are already sick and tired of it*” (I14, Cluj-Napoca).

In this context, art in the online environment is different in character, the most significant change being the absence of any emotion created by face-to-face contact between the supplier and the consumer of the artistic act: “*In an area like creativity (that of drama—note ours), online is a fake!*” (I6, Basca, Timișoara). This gives rise to a feeling, not always justified, that online alternatives are of doubtful and inferior quality and are incapable of satisfying consumer demand. The same hesitancy also applies to coworking spaces, where, with the exception of events, renting out a space as such is not seen by their managers as possible:

“*Since we are a physical/coworking space, somewhere we encourage people to come to, there is ZERO possibility of moving to online*” (I11, Timișoara).

## 4. Discussion

In terms of resilience [37] and social resilience in particular, the adaptation and survival potential of any particular cultural spaces, whether they be public or private entities, largely dependent on and even conditioned (as they are) by the decisions of national and local authorities, including regarding whether to reduce gaps between the funding of public bodies and of the private and independent sector [29], springs from the personal ability and flexibility of each artist or community of artists to reinvent themselves, of their social support, and of the entrepreneurial skills of the creatives concerned.

The degree to which artists have confidence in the government as their principal underlying logistical and financial supporter was and is directly proportional to the economic support they receive (financing schemes, thematic contests) and to the extent to which independent cultural happenings are promoted in the official media. In Romania, for the duration of the pandemic, we saw state involvement, through specialized organs (government, the ministry concerned, local authorities), in supporting publicly funded cultural institutions, frequently as a form of damage limitation. This did not cover independent artists who were not affiliated to any authorized and accredited institutions or associations. The immediate solution offered by the government was designed to provide an income for cultural workers, as follows: their full salary for public sector employees and 75% of the mean gross income as technical unemployment benefit in the private sector, in accordance with Emergency Government Ordinance 32/2020.

In the view of the independent artists we surveyed, the involvement of national and local authorities ought, in addition to this, to find expression in the short term in improving preventive healthcare, which impacts the entire population, and in the medium and longer term in encouraging different forms of cultural association (clusters, hubs), creating programs to fund cultural entrepreneurs and artist-in-residence schemes for young artists, rethinking government policy to emphasize culture, and even devising a way to provide independent artists in Romania with an official status.

As for the development perspectives of people working in the cultural sector, as of those who work in allied areas but support events involving various categories of artists, some interviewees once again make reference to different kinds of adaptation, such as:

- Developing businesses in the digital area, which is an option seen as viable by some of those interviewed (I4, I16, I11):
  “*In the future, digital arts, interactive arts, and online art will flourish. It is likely that artists will orient themselves more towards the digital and that this will develop*” (I4, Timișoara) and as less so by others (I1, Timișoara).
- Conceiving new cultural products that are experiential (I2, I5, I19) or even social in nature (Digital Canvas, Centrul de Interes, MindsHub);
- Rethinking the relationship with the consumer public through encouraging participative actions:
  “*We are thinking about whether we want to make an institution in which everyone acts, or something in the style of a rehearsal room*” (I25, Cluj-Napoca);
- A confidence that the cultural–artistic world follows a cyclical pattern of development, as seen in the encouragement of activities and events that imply physical interaction (I3, I4, I14, I22):
  “*We are social beings. More than that, I believe that after this online fever that is tending to lead to an extreme, there will be a bounce back that will bring us back together physically again. People will be hungry for direct interaction*” (I3, Casa cu Iederă, Timișoara).

The social resilience of the cultural sector in Romania, both public and private/independent, was shown in ways that differed from one city to the other, from one area of activity to another, and even from one individual to another, since it was conditioned by the factors of adaptability and solidarity. While forms of cultural adaptation have already been detailed, including by reproducing the opinions of the cultural community in view, where solidarity is concerned, this extended but also modified the forms of collaboration that existed before the pandemic. It is in fact clear that the level of social interaction and collaboration between those active in the cultural sector and comparable, similar bodies and even new dialogue partners was directly proportional to the depth and breadth of the collaborative relationships they had had in the period that preceded the pandemic. These kinds of community-type synergies, created ad hoc during the pandemic or building on previously existing partnerships to create clusters/hubs/networking platforms, outgrew the sphere of cultural–artistic activity and became relevant to society in general. We may give a number of examples to illustrate this:

- The creation of discussion groups of local actors that impact the development of the cultural sector (artists, businessmen, public institutions, local authorities, etc.) by implementing one-off measures, in a first phase (petitions, open letters), and subsequently by restructuring policy in the two cities;
- The mobilizing of artists and creative space managers to pool their efforts in order to benefit the health sector. Here, we may mention the OneSingleCluj platform, which, under the slogan “We are giving help and working together”, brought together representatives of the Cluj-Napoca cultural community to help administer a resource center, supported by the local authorities, whose beneficiaries were the city’s health institutions. Other examples of mutual aid may be seen in the contributions of the Artmedia foundation in Timișoara, which organized a Solidarity Marathon, and the Cluj Cultural Centre, which offered a Therapy through Art program under the auspices of the Art and Well-Being project.
- Consolidating a participative dialogue with the public, either in connection with a particular creative space or with a view to establishing new collaborative structures.

## 5. Conclusions

Our research project should be seen as part of the wider trend toward studying the socio-economic consequences of contemporary environmental challenges. While the fact that society is exposed to biological risks has long been recognized, the COVID-19 pandemic has placed it in a new context. This has been so since the first large-scale appearance, almost instantaneous and global in scale, of the reverberations and the consequences of this complex and almost all-encompassing phenomenon, with the inter-conditioned and hard-to-foresee dynamics of its various components.

These factors also account for the limitations of our research concerning the consequences of the COVID-19 crisis on the cultural and creative industries in the two cities selected as case studies:

- The empirical research was carried out for a segment of time (16 March–17 November 2020) and into a developing phenomenon;
- The qualitative research was focused on 25 independent cultural–creative spaces in Timișoara and Cluj-Napoca, and the results reflect the actions, perceptions, and plans of the businesspeople and administrators interviewed. Some findings could be extrapolated on the basis of a more general knowledge, derived from our previous research, of the amplitude and condition of this sector in the two cities and in Romania more generally.
- The degree of reactivity discovered is striking (only six of the 141 spaces/activities we surveyed had closed), but taking into account the extension of the restrictions even after the close of our investigation period, it is likely that their adapting/failing to adapt behaviors will also change.

Consequently, perspectives regarding the study of the resilience of the cultural and creative sector remain open. We can still see developments taking place in how those concerned are adapting to the restrictions, how businessman and creatives are finding organizational, technical, and creative ways forward, pressure being exerted on the authorities by actors in the area to make them aware of the needs, changes in the kinds of public support available, and so forth, in a situation in which social resilience is proving to have not only technical but also political aspects [23].

Another direction in the study and understanding of SCC resilience would involve looking at changes that have taken place among consumers of culture, whose behaviors have been influenced both by the restrictions and by the digital solutions to which they have had access and with which they have become familiar over this period. Some of these mutations may be an indication of long-term trends.

Therefore, it is quite conceivable that the pandemic may, where culture is concerned, prove to be a genuine evolutionary watershed across which culture travels from its classic mode of expression, in direct contact with the consumer, to a virtual one, the mechanism of this change being the large-scale involvement of technology in the transmission and rendering of its messages. This is a point that will need to be borne in mind by the cultural spaces of the future.

## Figures and Tables

**Table 1:** Features of ICCS structures in Timișoara and Cluj-Napoca in which interviews were conducted.

No. crt.	Denomination	Creative Space	Interview Date	City	Identification Code
1	INCUBOXX	Accelerators business	20 October 2020	Timișoara	I1
2	Multiplexity	Community space	26 October 2020	Timișoara	I2
3	Casa cu Iederă și Apiarium	Community space	27 October 2020	Timișoara	I3
4	Digital Canvas	Maker space	3 November 2020	Timișoara	I4
5	DIVE	Community space	3 November 2020	Timișoara	I5
6	Solid Arts and Entertainment și Basca	Community space	5 November 2020	Timișoara	I6
7	Lapsus	Maker space	9 October 2020	Timișoara	I7
8	Plan Zero	Maker space	4 November 2020	Timișoara	I8
9	TeMiriCE	Coworking space	18 October 2020	Timișoara	I9
10	Cowork Timișoara	Coworking space	16 November 2020	Timișoara	I10
11	MindsHub	Community space	5 November 2020	Timișoara	I11
12	Atelier Patru	Community space	15 October 2020	Cluj-Napoca	I12
13	Centrul de Interes	Community space	13 October 2020	Cluj-Napoca	I13
14	Create.act.enjoy	Community space	24 October 2020	Cluj-Napoca	I14
15	Loc în spațiu	Community space	13 October 2020	Cluj-Napoca	I15
16	Magic Puppet	Community space	13 October 2020	Cluj-Napoca	I16
17	Matca Space	Community space	29 October 2020	Cluj-Napoca	I17
18	Milestone	Coworking space	17 November 2020	Cluj-Napoca	I18
19	Music Hub	Community space	13 October 2020	Cluj-Napoca	I19
20	Muzeon	Community space	29 October 2020	Cluj-Napoca	I20
21	ParaPark	Community space	13 October 2020	Cluj-Napoca	I21
22	Reactor	Community space	20 October 2020	Cluj-Napoca	I22
23	RoCreator	Maker space	26 October 2020	Cluj-Napoca	I23
24	Smash Studio	Maker space	20 October 2020	Cluj-Napoca	I24
25	Zug.Zone	Community space	15 October 2020	Cluj-Napoca	I25

**Table 2:** SWOT Analysis.

Strengths	Weaknesses
Presence of advanced university centers (Bucharest, Cluj-Napoca, Timisoara, Iasi, Sibiu, Baia Mare, etc.), which provide cultural and creative professionals, enhanced by IT training programs The existence of a complex and diverse urban cultural agenda, public, private, or independent, multiplied by the existing cultural competitions in Timisoara (European Capital of Culture 2023) and Cluj-Napoca (European Youth Capital 2015) The assertion of cosmopolitan and dynamic urban environments in both cities The strong advocacy for tolerance, creativity, and participatory involvement of the civic community in the two investigated cities (Someș Delivery initiatives, La Terenuri Mănăștur, Intercultural Institute Timișoara, Heritage of Timișoara, etc.) Affirmation of cultural networks/platforms such as clusters (Clusterul Industrii Creative Transilvania Cluj-Napoca, MultipleXity Timișoara), hubs, cultural innovation parks (Faber Timișoara, CREIC Cluj-Napoca), international cultural centers (French Institute, British Cultural Center, German Cultural Center, etc.)	Precariousness of national and regional cultural and creative policies and programs, including those of cultural and participatory public education Vulnerability of the status of artists and cultural workers; status conditioned by the determined existence of contracts associated with events, shows Poor funding and the dependence of the cultural and creative sector on public funding Problems of transparency in the allocation of contracts in local competitions Lack of competent and dedicated cultural managers (local leadership) Volatility of the actual locations of the independent creative spaces in Cluj-Napoca and Timișoara (high rental price, temporary use of industrial platforms, restriction of some spaces, limited at a certain type of activity undertaken)
**Opportunities**	**Threats**
Supporting the European ICCS through support programs for cultural and creative activities but also complementary initiatives, such as the digital features Reinforcement of the creative and cultural activities through entrepreneurial and educational investments (workshops, courses) Rethinking the cultural policies and the cultural marketing strategies of relevant professionals, including increasing the public participation Introduction of new artistic and cultural curatorial approaches	Behavioral changes of the cultural consumer, including as a result of the psycho-social effects of the COVID-19 pandemic Degradation of the quality of the artistic and cultural act Decreasing the degree of physical interaction of artists and cultural workers with the public/consumer Increasing bureaucracy in the process of accessing cultural competitions

**Table 3:** Performance indices of cultural and creative industries in cities in Romania.

Index Performance	Bucharest	Cluj-Napoca	Timisoara	Iasi	Sibiu	Baia-Mare
Cultural and Creative Cities Index (total)	**22.8**	**20.5**	**18.1**	**16.1**	**18.2**	**14.2**
Cultural Vibrancy *	11.9	18.3	14.6	14.8	20.7	15
Creative Economy **	34.1	19.8	17.1	14.3	12.3	9.6
Enabling Environment ***	22	26.2	27.1	22.4	25.1	21.8

* Cultural Venues and Facilities; Cultural Participation and Attractiveness; ** Creative and Knowledge-based Jobs; Intellectual Property and Innovation; New Jobs in Creative Sectors; *** Human Capital and Education; Openness, Tolerance and Trust; Local and International Connections; Quality of Governance; Normalization: indicators range on a scale of 0 to 100; Source: Cultural and Creative Cities Monitor, 2019 [31].

## Data Availability

Not applicable.
